# Unveiling the Enigma: John Cunningham Virus-Associated Progressive Multifocal Leukoencephalopathy in an Immunocompetent Individual

**DOI:** 10.7759/cureus.64758

**Published:** 2024-07-17

**Authors:** Praveen Arumugam, Lovelina Singh, Puneet Agarwal, Suraj Shetti, Shalini Sharan

**Affiliations:** 1 Internal Medicine, Max Smart Super Speciality Hospital, Saket, New Delhi, IND; 2 Neurology, Max Smart Super Speciality Hospital, Saket, New Delhi, IND

**Keywords:** immunocompetent, mirtazapine, pml treatment, mefloquine, prognosis of progressive multifocal leukoencephalopathy (pml), progressive multifocal leukoencephalopathy (pml)

## Abstract

Progressive multifocal leukoencephalopathy (PML) is considered an often fatal, demon-leading disease primarily associated with immunosuppression. Immunocompromised individuals predominantly exhibit this manifestation, while immunocompatible patients rarely encounter it. We present a unique case of PML in an immunocompetent individual who initially presented with stroke-like symptoms, received management, and was subsequently discharged. He returned to our hospital a few days later with similar complaints, prompting further investigations that revealed PML, a condition often overlooked, especially in individuals with an intact immune system. Although he received successful treatment with mefloquine and other anti-malarial medications and followed up on an outpatient basis, his subsequent outcome was unfavourable. As a result, this case emphasises the importance of having PML as a significant differential and therapeutic option.

## Introduction

John Cunningham virus (JCV) causes progressive multifocal leukoencephalopathy (PML), a condition affecting the central nervous system. This deoxyribonucleic acid (DNA) virus belongs to the family *Papovaviridae*, genus *Polyomavirus*, BK virus, and SV 40 virus, which spreads through aerosols, oral-faecal route, blood transfusion or organ transplantation, person-to-person contact and remains dormant until the individual's immunity is challenged or weakened. The clinical manifestation of PML typically involves a gradual neurological impairment that manifests as ataxia, monoparesis, or hemiparesis. Contrary to its name, the illness affects more than just the cerebral white matter; it can also manifest as cortical abnormalities such as dysphasia, cortical blindness, or seizures [1].

We hereby report the presentation of PML in a non-immunocompromised patient, which initially manifested as a stroke but later manifested as PML on further investigation. While only a small percentage of immunocompetent patients with PML undergo spontaneous remission [2], this underscores the potential for patients to initially present with a localised impairment, typically treated like a stroke. Conducting an in-depth analysis is critical to avoiding improper or delayed treatment for the underlying causative factor. Examining the possibility of immuno-suppression is critical because there is a high probability of relapse due to an undetected loss in the cellular immune system.

## Case presentation

A male in his early 60s was a follow-up case for a recently diagnosed stroke on dual antiplatelets. He initially complained of right-sided weakness, which progressively worsened over the course of two months. The weakness manifested as difficulties in writing and performing fine movements, an imbalance when walking, and a tendency to lean more towards the right side. His family noticed a few instances of falling twice over the course of two to three months, and he gradually developed slurred speech, which manifested as difficulty verbalizing words despite his ability to comprehend. In addition, he experienced intermittent headaches, which were more localized to his back.

He denied any symptoms suggestive of loss of consciousness, vomiting, seizures, altered sensation, or bowel and bladder dysfunction. Although he was regular with neuro-rehabilitation, his symptoms worsened over time. His other comorbidities include a sleep apnea diagnosis three years ago, two years of self-discontinuation on bi-level positive airway pressure (BiPAP), underwent coronary artery bypass graft 17 years ago, and was treated for pulmonary tuberculosis.

**Figure 1:**
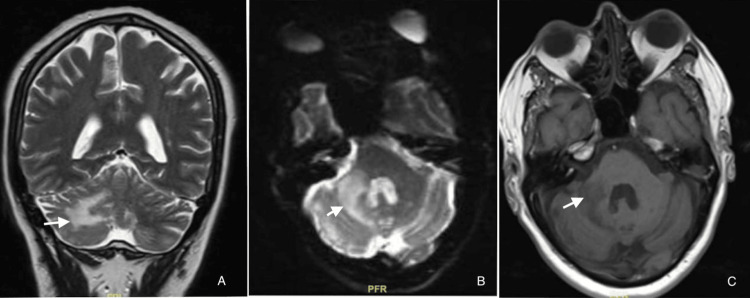
Baseline MRI images (A) MRI brain with a contrast, coronal section in T2 turbo spin echo (TSE) reveals hyperintensity (white arrow) in the right cerebellar hemisphere. (B) MRI brain echo planar 2D (EP2D) differential scan suggestive of hyperintensity (white arrow), in the right cerebellar hemisphere. (C) MRI brain T1-weighted image reveals a hypodense lesion in the right cerebellar hemisphere (white arrow), predominantly involving white matter.

Upon examination, he was conscious and oriented. The pupils exhibited normal size and were responsive to light on both sides, despite their impaired speech fluency and intact comprehension. His power in both upper and lower limbs was normal; his hand grip was weak bilaterally, and his deep tendon reflexes were normal, with the right side showing more positive Romberg's and past points than the left. His routine workup, including complete blood count, renal function, and liver function, all fell within normal limits upon further evaluation. His serology test revealed no evidence of human immunodeficiency virus (HIV) 1 and 2, and hepatitis C virus (HCV) and HbSAg tested negative, with the exception of a mildly elevated C-reactive protein. We performed an antinuclear antibody (ANA) to rule out any auto-immune conditions, but the results were normal. An MRI of the brain revealed a focal, poorly defined, patchy area of altered signal intensity in the right cerebellum. White matter dominated this area, and the right cerebellar peduncle adjacent to it showed focal involvement without any limited diffusion or blooming. The MRI angiogram revealed an increase in lesion size compared to the previous MRI done a few months ago due to a cerebral vascular accident (CVA). The CT angiogram of his neck revealed 75-80% stenosis in the right proximal internal carotid artery (ICA), over 90% at the right external carotid artery's (ECA) origin, 40-50% stenosis in the left proximal ICA, and 70-75% at the left ECA's origin. We subjected him to a bilaterally prolonged visual evoked potential. A second MRI brain plain and contrast (Figure 1) showed a confluent area of signal change in the right cerebellar hemisphere. This change was mostly in white matter and extended to the right-sided cerebellar vermis and the right middle cerebellar peduncle. This is likely due to the worsening symptoms and demyelination. There were a few small chronic ischemic foci in supratentorial white matter, but the spine is normal.

After the anti-platelet washout period, we scheduled a lumbar puncture for him, which revealed albumin cytological dissociation without cobwebs and no raised cerebrospinal fluid (CSF) total leukocyte count. We conducted further investigations to rule out the possibility of auto-immune encephalitis, which included anti-neuromyelitis optica (anti-NMO), aquaporin 4, anti-myelin oligodendrocyte glycoprotein (anti-MOG), fungal culture, cryptococcus-GeneXpert MTB, and Mycobacterium tuberculosis complex real-time polymerase chain reaction (PCR). All these tests were negative, except for the detection of JCV DNA in CSF, which suggested PML. Meanwhile, the ANA and Vasculitis Panel-LIA tested positive for polymyositis-scleroderma complex (PM-scl).

**Figure 2:**
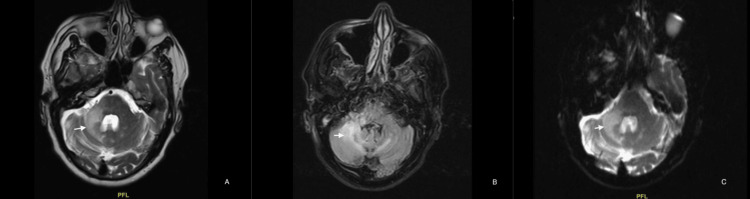
Follow-up MRI images (A) MRI brain with contrast shows hyperintensity (white arrow) in the right cerebellar hemisphere. (B) MRI brain FLAIR sequences show persistent hyperintensity (white arrow) in the right cerebellar hemisphere with a slight extension into the left cerebellar hemisphere as well. Although there were no interval radiological changes, the patient improved clinically. (C) MRI brain in the DWI sequence shows persistent hyperintensity (white arrow) in the right cerebellar hemisphere. FLAIR: fluid-attenuated inversion recovery; DWI: diffusion-weighted imaging

We started him on high-dose IV methylprednisolone at 1 mg/kg body weight, administering a total of five doses along with other supportive measures. Later, we started him on oral mirtazapine 7.5 mg once daily at night and mefloquine 250 mg once a week for four weeks, after which we discharged him and advised him to follow up on an outpatient basis. He showed significant neurological improvement over the second and fourth-week follow-ups, although a repeat MRI brain plain with contrast (Figure 2) done at the four-week follow-up did not show any significant radiological improvement.

Over the course of the following three months, he experienced repeated hospitalizations as a result of lower respiratory tract infections, leading to a steady deterioration in his overall health, which a multidisciplinary team cautiously addressed. Despite the best efforts and adequate measures involving a multi-disciplinary team, he was brought unconscious to the emergency department from home and was declared dead, with the cause of death likely due to a cardiac event.

## Discussion

Infection with the JCV is species-specific and exclusively occurs in humans. JCV infection, unlike other polyomavirus infections, has a limited host cell range. As a result, the lack of an animal model has impeded research on JCV pathogenesis. While all JCV-infected oligodendrocytes appear to be productive, some astrocytes do harbour late. Some astrocytes delete JCV genes, while others may have a failed infection and appear altered. Attached to the JCV, cells can incorporate an N-linked glycoprotein containing alpha (2,6)-linked sialic acid, among other things [3]. Many human cells contain this protein. Furthermore, in permissive astroglial cell cultures, the JCV can attach to the serotoninergic 5HT2a receptor. Pharmacologic drugs that target the 5HT2a receptor prevent infection of these cells in vitro [4]. Despite the widespread distribution of JCV receptors and the presence of JCV DNA in various cell types, including oligodendrocytes, astrocytes, lymphocytes, tonsil stromal cells, plasma cells, and kidney epithelial cells, success in cultivating JCV in human cell culture systems has proven elusive [5-7].

Individuals diagnosed with blood-related cancers, post-transplantation, and those receiving immuno-modulator drugs for auto-immune diseases also had increased incidences of PML [8-10]. Researchers have also discovered it in HIV patients who are on HAART (highly active antiretroviral therapy) medication [11]. In HIV-negative patients, it was observed that the median survival time was three months as per Koralnik [12].

As there are various causes of immunosuppression, one such could be cell-mediated immunity, especially CD4+ cells, which plays an important role in the suppression of the JCV. Though the CD4+ and CD8+ counts are normal, deficiencies in the immune response may allow PML to develop. Cirrhosis was discovered in seven PML patients in one analysis of 38 reported cases of PML in individuals with probable occult or transitory immunosuppression by Gheuens et al. [13]. Out of which, three of them were observed to have a low CD4+ lymphocyte count. Patients with hepatitis C or hepatitis B liver illness had a significant decrease in cell-mediated immune function, particularly in the presence of low haemoglobin and albumin [14].

Prior cases of PML in patients with low JCV viral load have been recorded, as low viral load does not correlate with illness severity. When compared to brain biopsy for individuals with HIV-associated PML, PCR detection of JCV DNA in the CSF has been demonstrated to have a specificity of 92% to 100% and a sensitivity of 74% to 92% [15,16]. It is assumed that any level of virus in the CSF should be considered problematic for PML, with a range of undetectable to 7.71 log copies/mL observed in patients with PML in a previous study by Bossolasco et al. [16].

It is important to understand that the virus's DNA is prevalent in the brains of healthy humans, and this viral load seems to rise in immunocompromised conditions, as observed in an autopsy study of HIV patients without PML (no clinical symptoms during life and no neuropathologic evidence of PML) as per the study released in 2013 by Bayliss et al. [17]. However, the expression of viral proteins, as revealed by immunohistochemistry, is considered an unequivocal marker of this disease. In their case, viral DNA was discovered in the biopsy material, but viral protein expression was not picked out [17]. As a result, PML was not initially thought to be the most likely diagnosis in this immunocompetent patient. Identifying the parameters that influence JCV reactivation in an immunocompetent patient will make a significant contribution to our understanding of PML pathophysiology.

There was a study that assessed the outcome of mefloquine treatment on PML and characteristics that may predict PML outcomes based on an in vitro study. This 38-week open-label randomised, parallel-group proof-of-concept trial compared patients with PML who got standard of care (SOC) with those who were administered the SOC with mefloquine (250 mg for three days, followed by 250 mg monthly). Patients assigned to SOC might begin mefloquine medication at week four. The main goal was the change in JCV DNA viral load in CSF from baseline to the fourth and eighth weeks. A few exploratory studies looked for parameters that could be related to clinical outcomes. A high proportion of individuals enrolled were positive for HIV. Although pre-planned interim data analysis indicated that the study was unconvincing to effectively establish a considerable distinctness between the groups, the trial was discontinued prematurely. With regards to JCV DNA levels or clinical or MRI findings, there was no analytical distinction [18]. In our patient, we followed a similar regimen, but the outcome was unfavourable, as there was significant clinical improvement, albeit due to a cardiac event while doing his daily chores.

Mefloquine, an antimalarial drug, was demonstrated to be superior in lowering the concentrations of viral load levels in CSF in an experimental investigation. The drug mefloquine did not hinder the viral load in the CSF, as per Freidman [19]. However, out of 24 individuals studied, there were only three HIV-negative patients, while the rest were all HIV-positive and were on HAART. There was no undisputable evidence, as the number of patients recruited without AIDS-related PML like auto-immune conditions or malignancy was negligible. Furthermore, while we employed monotherapy in this clinical trial, we also used a combination treatment of mefloquine and mirtazapine, inspired by a study by Eperla et al. [20]. Compared to radiological progression, our patient's functional and cognitive responses got better after starting multimodal therapy with mirtazapine and mefloquine. This suggests that randomised controlled trials in people with non-immunocompromised PML are needed to confirm this treatment strategy.

## Conclusions

This case report highlights a rare instance of PML occurring in an immunocompetent individual, although he was successfully managed with combination therapy of mefloquine and mirtazapine as there was a significant improvement in his clinical condition, despite no interval change in radiologically. This helps us look for alternative approaches for immunocompetent patients with PML. In this patient, the overall outcome was inauspicious due to a cardiac event, which puts us in the dilemma of determining whether the cause of death is probably due to his comorbidities or the progression of the disease despite clinical improvement. This case emphasises the significance of having PML in differential diagnoses, even in immunocompetent patients, and exploring innovative treatment strategies for this traditionally difficult-to-treat condition. More additional clinical trials or studies are necessary to check the safety and efficacy of this combination therapy, especially in larger population groups.
